# The Obstetric Catechism; Containing 2347 Questions and Answers on Obstetrics Proper

**Published:** 1860-01

**Authors:** 


Art. V.—*The Obstetric Catechism; containing* 2347 *Questions and Answers on Obstetrics Proper.* By Joseph Warrington, M.D. With 150 Illustrations. 12mo., pp. 445. J. B. Lippincott & Co.: Philadelphia, 1860.

We presume this to be a new edition of this work, although the fact is not stated on the title-page, where one would naturally look for such an announcement. The first edition appeared in 1853. As a general rule, we have no special fancy for manuals of this description, but we may readily make an exception in favor of the present production, knowing it to be the offspring of a man of sound sense, of large experience, and of no little practical tact; the author having for many years been at the head of the Philadelphia Obstetric Institute, a charity affording ample opportunities for observation. The work is very appropriately dedicated by Dr. Warrington to his pupils, and is written with great modesty, but accuracy and clearness. The wood-cuts are, for the most part, miserably destitute of artistic finish.

